# The Efficient Derivation of Trophoblast Cells from *Porcine In Vitro* Fertilized and Parthenogenetic Blastocysts and Culture with ROCK Inhibitor Y-27632

**DOI:** 10.1371/journal.pone.0142442

**Published:** 2015-11-10

**Authors:** Dongxia Hou, Min Su, Xiawei Li, Zhiying Li, Ting Yun, Yuhang Zhao, Manling Zhang, Lihua Zhao, Rongfeng Li, Haiquan Yu, Xueling Li

**Affiliations:** 1 The Key Laboratory of National Education Ministry for Mammalian Reproductive Biology and Biotechnology, Inner Mongolia University, Hohhot, Inner Mongolia, P. R. China; 2 State Key Laboratories of Reproductive Medicine, Nanjing Medical University, Nanjing, China; 3 Jiangsu Key Laboratory of Xenotransplantation, Nanjing Medical University, Nanjing, China; Michigan State University, UNITED STATES

## Abstract

Trophoblasts (TR) are specialized cells of the placenta and play an important role in embryo implantation. The *in vitro* culture of trophoblasts provided an important tool to investigate the mechanisms of implantation. In the present study, porcine trophoblast cells were derived from pig *in vitro* fertilized (IVF) and parthenogenetically activated (PA) blastocysts via culturing in medium supplemented with KnockOut serum replacement (KOSR) and basic fibroblast growth factor (bFGF) on STO feeder layers, and the effect of ROCK (Rho-associated coiled-coil protein kinases) inhibiter Y-27632 on the cell lines culture was tested. 5 PA blastocyst derived cell lines and 2 IVF blastocyst derived cell lines have been cultured more than 20 passages; one PA cell lines reached 110 passages without obvious morphological alteration. The derived trophoblast cells exhibited epithelium-like morphology, rich in lipid droplets, and had obvious defined boundaries with the feeder cells. The cells were histochemically stained positive for alkaline phosphatase. The expression of TR lineage markers, such as CDX2, KRT7, KRT18, *TEAD4*, *ELF5* and *HAND1*, imprinted genes such as *IGF2*, *PEG1* and *PEG10*, and telomerase activity related genes *TERC* and *TERF2* were detected by immunofluorescence staining, reverse transcription PCR and quantitative real-time PCR analyses. Both PA and IVF blastocysts derived trophoblast cells possessed the ability to differentiate into mature trophoblast cells *in vitro*. The addition of Y-27632 improved the growth of both PA and IVF blastocyst derived cell lines and increased the expression of trophoblast genes. This study has provided an alternative highly efficient method to establish trophoblast for research focused on peri-implantation and placenta development in IVF and PA embryos.

## Introduction

Porcine embryos can be produced *in vitro* by different technology, such as *in vitro* fertilization (IVF), somatic cell nuclear transfer (SCNT), and parthenogenetic activation (PA). The derived embryos are important for agriculture and biomedical research [1]. However, these *in vitro* produced embryos are less developmentally competent than *in vivo*-derived embryos, especially for the PA derived embryos. Porcine mature oocytes can be parthenogenetically activated by various methods [2, 3], and the derived embryos are valuable resources for studying the gene imprinting [4], and a potential alternative source of derivation of primed embryonic stem cells [4, 5]. However, parthenogenetically derived blastocysts have a reduced total cell count and fewer cells in the inner cell mass (ICM) compared to fertilized embryos [6–8], and they usually experience severe developmental failure [9, 10]. Although, the PA embryos can develop to the morulae or blastocysts *in vitro* [2, 11–13], they stop developing at different stages of gestation [14, 15] *in vivo*. The mechanisms underlying the deficiencies of embryos generated from PA and SCNT have not been completely understood [16].

The trophectoderm (TE) of the pre-implantation blastocyst is considered as the first differentiated tissue [17, 18]. Trophoblasts (TR) will differentiate into the fetal part of placenta during development; therefore, these cells can be used to investigate trophectoderm differentiation and placental development. However, the trophoblast development mechanism behind the deficiencies of embryos generated from PA are not completely understood and *in vitro* studies of the role of porcine PA trophoblasts in the maintenance of pregnancy have been hindered due to difficulties in obtaining pure populations of non-transformed trophoblastic cells [19]. Several porcine trophoblast cell lines have been described previously, such as the Jag1 [20], TE1 [19], TBA [21] and iTR [22] lines, but the reports on derivation and characterization of parthenogenetically derived trophoblast cells are rare, except Saadeldin et al. who recently reported that the post-maturation zona perforation of oocytes improved porcine parthenogenetic trophoblast cultures [23]. These porcine trophoblast cells were derived from Day 9, 14 and 15 pre-implantation porcine embryos [19–21], while iTR was derived during reprogramming of porcine mesenchymal cells with a four-factor (POU5F1/SOX2/KLF4/MYC) mixture of vectors [22]. All these pig trophoblasts have the capacity to spontaneously grow in culture and, in the absence of any immortalization procedure, reach high passage numbers while retaining its characterization [21]. The cells display epithelial characteristics, produce selected cytokines (IFND, IFNG, and IL1B) [20–23]. However the trophoblast related marker gene expression such as *CDX2*, *HAND1*, and *ELF5* is only analyzed on iTR cells [22].

Dulbecco's modified eagle medium (DMEM) supplemented with fetal bovine serum (FBS) is the common trophoblast cells culturing medium, while Dulbecco's modified eagle medium: Nutrient mixture F-12 (DMEM/F12) with KnockOut serum replacement (KOSR) and basic fibroblast growth factor (bFGF) are usually used to culture embryonic stem cells. However, when porcine mesenchymal cells, whether from fetal connective tissue or from the umbilical cord, were subjected to standard reprogramming protocols, a significant fraction of the emergent colonies cultured on KOSR/bFGF media had features of TR [23].

Rho-associated coiled-coil protein kinases (ROCKs) are downstream effectors of the Rho GTPases, which include RhoA, Rac1, and CDC42 and regulate trophectoderm differentiation, cell polarity [24] and E-cadherin expression in cleavage stage embryos and a variety of other cell types [25, 26]. Y-27632 is known, as a highly selective ROCK inhibitor [27, 28], releases cell contractions [29] and maintains the pluripotency of stem cells [30]. Presence of 20μM Y-27632 increased the rate of attachment and differentiation of trophoblast differentiation from the hESCs [31]. Y-27632 inhibits the RhoA, Rho Kinases, MLC kinase pathway, and activate the alternative CDC42 and Rac pathways. These molecules are well known for their role in trophoblast cell migration, cell polarity determination and in epithelial mesenchymal transitions [32]. But the effect of ROCK inhibitor Y-27632 on *in vitro* cultured trophoblast has not been investigated so far.

In the present study, we seeded both IVF and PA derived porcine blastocysts into KOSR/bFGF culture system followed by Y-27632 supplement, in order to find the efficient culture system for trophoblasts from IVF and PA embryos, and investigate the effect of ROCK inhibitor on trophoblast growth and characteristics. More than 40% attached blastocysts could successfully grow and 30% outgrowths passaged more than 20 times. The addition of ROCK inhibitor Y-27632 improved the growth of derived cells and increased the expression of trophoblast genes. These cells were cytokeratin 7 (KRT7) and cytokeratin 18 (KRT18) positive and immunostained positive for CDX2 and stage specific embryonic antigen 1 (SSEA1). The expression of several trophoblast and imprinted genes was significantly different in PA TR cells and IVF TR cells, such as *CDX2* and *ELF5* were highly expressed in PA TR cells than in IVF TR cells, while *PEG1* and *PEG10* were highly expressed in IVF TR cells. Moreover, the trophoblast cells derived from PA and IVF blastocysts possess the ability to differentiate into mature trophoblast cells in DMEM/FBS medium or KOSR medium without bFGF.

## Materials and Methods

### Chemicals

All chemicals were purchased from Sigma-Aldrich, unless otherwise indicated.

### Animal care and use

All experiments with animals were conducted in accordance with the Guide for Care and Use of Laboratory Research Animals and were approved by Inner Mongolia University’s Animal Care and Use Committee.

### Porcine embryo culture and TR cell derivation

#### Oocyte collection and *in vitro* maturation (IVM)

Ovaries were collected at a local abattoir, transported in 0.9% (w/v) saline solution and then washed with saline solution upon arrival at the laboratory. Cumulus oocyte complexes (COCs) were collected from 3- to 6-mm diameter follicles of porcine ovaries with an 18-gauge needle and washed several times in PVA-TL HEPES buffer. Oocytes, as observed by stereo microscope, surrounded by more than two layers of cumulus cells were cultured in IVM medium (M199; Gibco, 11150), with 0.1% (w/v) PVA, 3.05 mM D-glucose, 0.91 mM sodium pyruvate, 0.57 mM cysteine, 0.5 mg/mL luteotropic hormone (LH; L-5269), 0.5 mg/mL follicle-stimulating hormone (FSH; F-2293), 10 ng/mL epidermal growth factor (EGF; Promega, G5021), 75 mg/mL penicillin G, and 50 mg/mL streptomycin). Groups of 100 oocytes were transferred to individual well of a four-well plate (Nalge Nunc International, Rochester, NY, USA) containing 700 μL IVM medium. The wells were subsequently covered with mineral oil and incubated at 38.5°C in a 5% CO_2_ atmosphere in air. After 42–44 hrs of maturation culture, the cumulus cells were removed by gently pipetting in PVA-TL HEPES buffer containing 0.1% hyaluronidase. Denuded oocytes with a visible first polar body and normal morphology were selected for further study.

#### Parthenogenetic activation (PA), *in vitro* fertilization (IVF) and embryo culture

Oocyte parthenogenetic activation was conducted by electrical stimulation. Oocytes were rinsed with warmed activation medium (0.3 M mannitol, 1.0 mM CaCl_2_·H_2_O, 0.1 mM MgCl_2_·6H_2_O and 0.5 mM HEPES in distilled water) and then aligned in a chamber with two electrodes 0.5 mm apart and covered with activation medium. Electrical activation was performed using two 30μs electrical pulses of 1.2 kV/cm. The activated oocytes were transferred in PZM-3 medium (108.0 mM NaCl, 10.0 mM KCl, 0.35 mM KH_2_PO4, 0.4 mM MgSO_4_·7H_2_O, 25.07 mM NaHCO_3_, 0.2 mM Na-pyruvate, 2.0 mM Ca(lactate)_2_·5H_2_O, 1.0 mM glutamine, 5.0 mM hypotaurine, 20 mL/L Eagle basal medium amino acid solution, 10 mL/L modified Eagle’s medium amino acid solution, 0.05 mg/mL gentamicin and 3 mg/mL BSA), and cultured for 6–8 days at 38.5°C, 5% CO_2_ in humidified air.

For IVF, every 40 matured oocytes were transferred to 50 μL drops of modified Tris-buffered medium (mTBM, with 113.1 mM NaCl, 3 mM KCl, 7.5 mM CaCl_2_N_2_·H_2_O, 20 mM Tris (crystallized free base; Fisher Scientific, Fair Lawn, NJ), 11 mM glucose, 5 mM sodium pyruvate, and no antibiotics). Ejaculated fresh porcine semen was diluted 3–5 times with sperm diluent. Two milliliters of diluted semen was added to 10 mL warmed sperm wash Dulbecco's phosphate buffered saline (DPBS) supplemented with 1 mg/mL BSA, 75 μg/mL penicillin, and 50 μg/mL streptomycin), and centrifuged (600×g, 8 min). The sperm pellet was suspended and added to the 50μL oocytes drops at a final concentration of 1×10^6^ sperm/mL. After 4 hrs, Putative zygotes were transferred to PZM-3 medium and cultured for 6–8 days at 38.5°C in a humidified gas environment of 5% CO_2_. Every 70–100 oocytes were cultured in 500 μL PZM-3 and the culture wells were covered with mineral oil. After 48 hrs of culture, the cleavage was examined. The blastocysts were used for porcine trophoblast cells derivation, ICM and TE differential staining and RNA extraction.

#### Embryo quality evaluation

For the differential staining, the expanded day 6–7 blastocysts were treated with 0.5% pronase to remove the zona pellucida, which was followed by exposure to rabbit anti-porcine whole serum (Sigma, B3759) at a 1:4 dilution in PZM-3 for 30min. Then, the blastocysts were rinsed in PZM-3 and placed into a 1:9 dilution in PZM-3 of guinea pig complement (Sigma, S1639), which contained 5 mg/mL propidium iodide and 40 mg/mL Hoechst 33342 for 15 min. The blastocysts were rinsed in DPBS (with 0.1% BSA) and mounted on glass slides, which were observed using an epifluorescence microscope (Ti-S; Nikon, Japan). Blue and pink cells were designated as ICM and TE cells, respectively.

#### Feeder cell preparation

STO cells used as feeders were purchased from Chinese Academy of Sciences cell bank (Shanghai). Cells were cultured in DMEM/F-12 (Gibco, 11330) plus 10% FBS (Hyclone, NWG0445) in 10 cm flasks. After treated with mitomycin C (15 mg/mL; Haizheng) for 2.5–3 h at 37°C, STO cells were washed thrice with phosphate buffered saline (PBS), trypsinized and frozen in FBS with 10% DMSO. Feeder cells were plated at 1.6×10^5^ /well in four-well culture plates (Nunc, Thermo) before TR cells were seeded.

#### Pig TR cell derivation

The PA and IVF blastocysts were incubated in 0.5% pronase for 1–2 min to eliminate zona pellucida. The zona-free whole blastocysts were transferred onto the mitomycin C-inactivated STO fibroblast feeder layers and cultured at 38.5°C in a humidified environment of 5% CO_2_ in air in the medium of DMEM/F12 (Gibco, 11330) supplemented with 20% KOSR (Gibco, N10828), 0.1 mM β-mercaptoethanol, 0.1 mM non-essential amino acids (Gibco, 11140), 1% (v/v) penicillin-streptomycin solution, and 20 ng/mL human recombination basic FGF (Promega, G5071), named KF medium. After 3–5 days, cells began to grow out from the blastocysts. The initial outgrowths was designated as passage zero (P0). The primary outgrowths were passaged to four-well plates with fresh media and new STO feeder layers by mechanical dissection into clumps under a stereo microscope. The growth of colonies was evaluated on a daily basis, with the cells passaged mechanically every 7 days, and the medium changed daily. According to the order in which they were derived from blastocysts, the cell lines were named pPATR1-11 for the electrically activated PA embryos and pIVFTR1-11 for the IVF embryos.

### Characterization of pig TR cells

#### Alkaline phosphatase (AP) Staining

The AP activity of the cells was detected with an AP detection kit (Promega, S371) according to the manufacturer’s instructions. Briefly, to every 5 mL of alkaline phosphatase buffer, 33 μL NBT and 16.5 μL BICP were added. Cells were fixed in 4% (w/v) paraformaldehyde for 1 min at room temperature, and then stained with the AP buffer for an additional 30 min-1 hr. The cells were washed with DPBS, and analyzed using a Nikon Ti-S microscope.

#### Immunocytochemistry

The blastocysts and pTR cells were both used for immunocytochemistry analysis. The cells were grown on 13-mm round coverslips covered by STO feeder cells. The blastocysts and pTR cells were briefly washed in DPBS and fixed in 4% paraformaldehyde for 10 min at room temperature. After three times of washing in DPBS, they were exposed to 1% Triton X-100 for 15 min to permeate the membranes. After washed with DPBS, the blastocysts and pTR cells were blocked with 10% goat serum for 1 hr at 37°C. The primary rabbit or mouse antibodies directed against OCT4 (Santa Cruz Biotechnology), SOX2 (Millipore), NANOG (Abcam), SSEA1 (Millipore), SSEA4(Millipore), TRA-1-60(Millipore), TRA-1-81(Millipore), CDX2 (Biogenex), KRT7 (DAKO) or KRT18 (FITC-conjugated, Sigma) were added at a 1:100 dilution in 10% goat serum, and incubated overnight at 4°C. For SSEA1, SSEA4, TRA-1-60, and TRA-1-81 staining, the permeabilization step was omitted. Afterward, the blastocysts and pTR cells were washed with DPBS and incubated with secondary antibodies Alexa 546-conjugated goat anti-rabbit immunoglobulin G (IgG), and Alexa 488-conjugated goat anti-mouse IgG (both from Invitrogen) in the dark at a 1:300 dilution for 1 hr at room temperature. Nuclei were stained with 10 ng/mL DAPI (4', 6-diamidino-2-phenylindole). Cells were then washed with DPBS and mounted onto slides for epifluorescent image acquisition with an Olympus (Germany) or Nikon C1 (Japan) confocal microscope.

#### Oil red O staining

The pTR cells from pig blastocysts were rich in lipid droplets. Their accumulation was evaluated by Oil Red O staining. Briefly, the 4-well plate-cultured cells were washed with DPBS, fixed for 30 min with 50% (v/v) ethyl alcohol, stained with 5 μg/mL Oil Red O (Sigma, USA) for 10 min, and counterstained with hematoxylin (Sigma, USA) for 30 sec. Finally, the cells were examined by light microscopy.

### Gene expression analysis

#### Reverse transcription polymerase chain reaction (RT-PCR)

Total RNA from the pTR cell lines, porcine embryonic fibroblasts (PFF), feeder cells and blastocysts was extracted using mRNA DIRECT Micro Kits (QIAGEN) according to the manufacturer’s instructions. RNA quality and quantity were determined using a NanoDrop 2000c Spectrophotometer (Thermo Scientific). The cDNA was obtained using PrimeScript^™^ RT reagent Kits (TAKARA). The reactions proceeded at 37°C for 15 min, followed by 85°C for 5 seconds, and the resultant products were stored at 4°C until they were analyzed. The primer sets used are listed in Table 1. For RT-PCR, cDNA amplification was performed in 20 μL reaction volumes containing 2 μL 10× DNA polymerase reaction buffer, 2 μL dNTP Mixture (10 mM), 1 μL (10 μM) each of the forward and reverse primers, 1 μL sample, 0.2 μL ExTaq DNA Polymerase (TAKARA), and 12.8 μL H_2_O. PCR amplification was performed using Applied Biosystems Thermo Cycler. The expression of porcine *β-ACTIN* was used as a control. All PCR reactions were initiated at 95°C for 3 min, followed by 35 cycles of 94°C for 15 sec, 60°C for the *β-ACTIN*, *ESRRB*, *SMARCA4*, *PAG* and *PL* genes, 58°C for the *OCT4*, *CDX2*, *TEAD4*, *ELF5*, *HAND1*, *CDH3*, *GATA3*, *ETS2* and *MASH2* genes for 15 sec and 72°C for 30 sec. Reactions were terminated around 10 min at 72°C. PCR products were resolved on a 1.5% agarose gel and imaged using a ChemiDoc XRS3 Molecular Imager (Bio-Rad).

**Table 1:** Primers used for Reverse Transcription Polymerase Chain Reaction and Quantative Real- time Polymerase Chain Reaction.

Gene	Forward Primers	Reverse Primers
β-ACTIN	GTGGACATCAGGAAGGACCTCTA	GTGGACATCAGGAAGGACCTCTA
OCT-4	GCTGACAACAACGAGAATCTGC	ACGCGGACCACATCCTTCTCTAG
CDX2	GGAGCTGGAGAAGGAGTTTCA	TGCAACTTCTTCTTGTTGATTTTC
TEAD4	CATGATCATCACCTGCTCCA	AAGTTCTCCAGCACGCTGTT
ESRRB	TCAAGGTGGAGAAGGAGGAG	TCCAGCATCTCCAGGAAGAG
ELF5	GCTTGAAAACAAGTGGCATC	TCTTCCTTTGTCCCCACATC
HAND1	ACATCGCCTACCTGATGGAC	TAACTCCAGCGCCCAGACT
CDH3	GTGCTGCCTGGCACTTCAGTGA	AACATGAGGTCGTGCGGGTCCT
GATA3	GAAGGCATCCAGACCAGAAA	CAGCATGTGGCTGGAGTG
ETS2	CGACAAGAACATCATCCACAAG	GATGGCGTGCAGTTCCTC
MASH2	GTGCCGCACCAGAACTCGTA	CAGCTTCTTGTTGGCGCCGC
SMARCA4	CTGACCTGTGAGGAGGAGGA	GCCTTGAGCCACTGCTTCT
PAG	ACCTCAAGTGGGTGCCCC	CAGGCCAATCCTGTTCTGTCC
IGF2	AAGAGTGCTCTTCCGTAG	TGTCATAGCGGAAGAACTTG
PEG1	TGTCATAGCGGAAGAACTTG	GGTGGACTTTGTGAGAGAG
PEG10	GTTGTTAATGGCTGGAAGAG	AGTCACTTCCCCTTCCTAAG
TERC	TACCGCCATCCACCATCCAG	TCTTCACGGCGGCAATGGAC
TERF2	TTCAGGCAGCACCAGATGAAG	TACTTCTGCACTCCAGCCTTG
GAPDH	TGTTGTGGATCTGACCTGCC	TGTCGTACCAGGAAATGAGCTT

#### Quantitative real-time PCR

Real-time PCR amplification was conducted using a Jena qTOWER 2.2 real-time PCR System (Germany). A KAPA SYBR FAST Universal qPCR Kit (KAPA Bio systems) was used to provide real-time quantification of desired PCR products according to the manufacturer’s instructions. Each real-time PCR reaction mixture contained 2 μL cDNA and 0.4 μL of each primer (Table 1) in a total volume of 20 μL. All tests were conducted in triplicate and product identity was confirmed by gel electrophoresis and melting curve analysis. The mRNA levels of trophoblasts genes and imprinted genes were normalized to that of mRNA encoding *β-ACTIN*; telomerase RNA component (*TERC*) and telomere-repeat binding factors 2 (*TERF2*) were normalized to that of mRNA encoding *GAPDH*.

### Chromosomal spread analysis

The chromosome numbers of established pTR cell lines from PA and IVF blastocysts were analyzed. Cells were incubated in KF medium supplemented with 0.02 μg/mL colcemide (Gibco) for 3 hrs at 38.5°C in an atmosphere of 5% CO_2_ in air. After trypsinization, cells were incubated in hypotonic KCl (0.56%, m/v) at 38.5°C for 15 min, then fixed in a mixture of methanol and acetic acid (3:1, v/v) and spread on clean microscopic slides by gentle dropping. After staining with Giemsa (1:20 dilution; Sigma) for 20 min, the chromosomes were examined at ×1000 magnification under a Nikon ECLIPSE 80i microscope.

### Culture of pTR cells with ROCK inhibitor Y-27632

The pTR cells were passaged mechanically as usual or separated into single cells by 0.25% trypsin-EDTA, then cultured in the KF medium supplemented with 10 μM Y-27632. After 48 h, Y-27632 was either removed from or remained in the media for the entire culture process. Cell line pPATR-5 and pIVFTR-7 were used to test the effect of Y-27632. The cell growth rate with or without Y-27632 was assayed by measuring the surface area of the colonies and counting the cell of each well over a 16-day period at 4-day intervals. PFF was used as control. The gene expression changes of pPATR-5 and pIVFTR-7 cells after cultured with Y-27632 were analyzed by real-time PCR.

### Western blot analysis

pTR cells cultured with or without Y-27632 were washed twice with ice-cold DPBS and lysed on ice for 5 min in extraction buffer (Thermo, 78503) containing protease inhibitors. The lysates were centrifuged at 14,000g for 10 min at 4°C, and the supernatant was collected. The protein content was determined by Pierce BCA protein assay (Thermo, 23225). The lysates (20ng protein) were separated by SDS-PAGE (4%~20%) and transferred to polyvinyldene fluoride membrane (Millipore). The membranes were blocked with 5% (w/v) skim milk and 0.5% (v/v) Tween-20 in Tris-buffered saline and subsequently incubated with primary antibodies directed against CDX2 (Biogenex), KRT7 (DAKO), KRT18 (Sigma) and ROCK2 (Abcom) at 1:1000 dilution, and β-ACTIN (Abcom) at 1:5000 dilution (Abcom) overnight at 4°C. The membranes were next washed in Tris-buffered saline with 0.5% (v/v) Tween-20 followed by incubation with peroxidase conjugated secondary antibody (at 1:2500 dilution; goat-anti-mouse, goat-anti-rabbit; Promega) for 45 min. The bands were detected by chemiluminescence signals using ECL Western Blotting Substrate (Promega, W1001) and scanned to produce digital images. The β-ACTIN band served as a standard.

### Culture of pTR cells with FBS or without bFGF

Two different media were used to test whether pTR cells could mature to later stage trophoblast cells. They are the high glucose DMEM supplement with 10% FBS (Hyclone, NWG0445) and 1mM glutamine and KF medium without bFGF. pTR cells that were mechanically cut under a microscope were cultured in the conditions that were mentioned above on 0.2% gelatin-coated dishes. The cells were cultured at 38.5°C for one or two week, and the medium was changed every 3 days. Additionally, on day7 and day14, total RNA was isolated from cells using an RNeasy Mini kit (QIAGEN) according to the manufacturer’s protocol. Reverse transcript PCR was performed to detect the gene expressions of cells cultured in different medium.

### Statistical Analysis

Statistical analyses were performed with SPSS19.0. Data are presented as means ± SEM as shown in the figures and tables. P values were calculated by t-test for comparisons of two groups and analysis of variance (ANOVA) for multiple pairwise comparisons. Differences were considered statistically significant when P< 0.05.

## Results

### Embryo culture and identification of blastocyst quality

**Fig 1:**
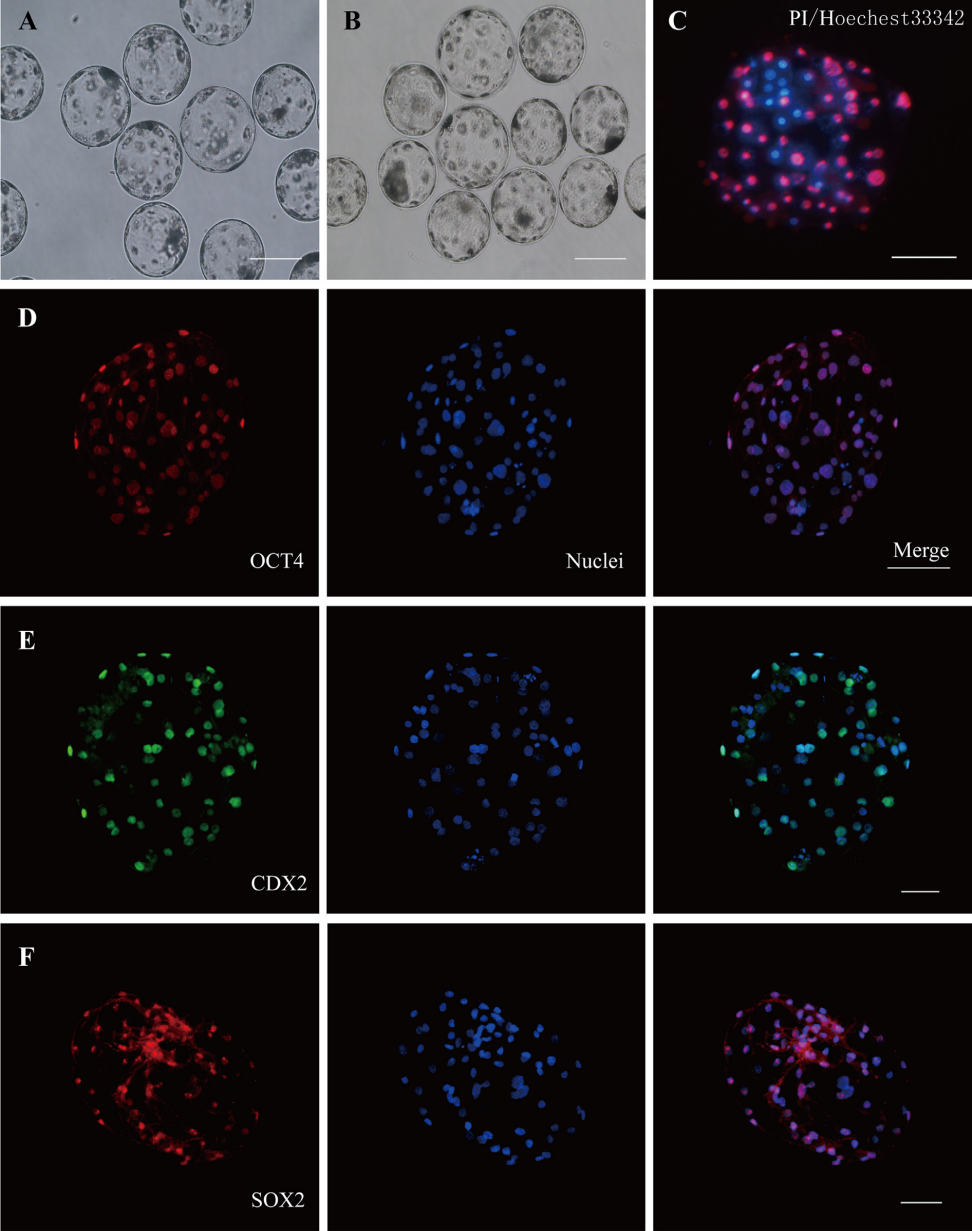
Porcine IVF and PA blastocysts quality identification. (A-B) Morphology of IVF blastocysts (A) and PA Blastocysts (B) at day 6. (C) Dual differential staining of porcine blastocyst, blue nucleus stained by hoechest33342 are designated as ICM cells, pink nucleus stained by both hoechest33342 and PI are designated as TE cells. (D-F) Immunofluorescence staining of pluripotency and trophoblast stem cells markers of porcine blastocysts. IVF blastocysts are OCT4 (D), CDX2 (E) and SOX2 (F) positive. There were no staining differences between IVF and PA blastocysts (S1 Fig). All scale bars represent 100μm.

Under similar conditions, a duplicate experiment was conducted with a total of 3221 mature porcine oocytes for parthenogenetic activation and *in vitro* fertilization. After electrical activation or fertilization, oocyte cleavage (2-cells) occurred within 48 hrs, while 4-cell and 8-cell embryos appeared within 3 days. The morulas commenced blastula at approximately 4 to 5 days. Most blastocysts appeared at day 6–7 with fluid accumulating in the blastocoele cavity, and inner cell mass apparent (Fig 1A and 1B). The ratio of cleavage and blastocyst formation was not significantly different between PA and IVF (Table 2). Dual differential staining was employed to distinguish ICM and TE cells. Blastocyst quality was evaluated by counting the number of ICM, TE, and whole blastocyst cells (Fig 1C). 19 IVF blastocysts from four replicates and 9 PA blastocysts from three replicates were analyzed (Table 3). The numbers of total cells in IVF blastocysts were significantly more than that in PA blastocysts (71.24±5.48 vs. 53.30±5.85, P< 0.05). The numbers of ICM cell (18.0±3.0 vs. 10.9±2.3) and TE cell (53.3±4.5 vs. 42.7±4.9) in IVF and PA blastocysts were not significantly different. However, the IVF blastocysts had higher ratio in ICM/Total (IVF vs. PA, 24.78±2.98 vs. 20.38±3.04, P>0.05), whereas PA blastocysts had higher ratio in TE/Total (PA vs. IVF, 79.62±3.04 vs. 75.22±2.98, P>0.05). These results demonstrated that IVF blastocysts not only contained more cells but also contained more ICM cells, whereas PA blastocysts had more TE cells. Immunofluorescence staining of OCT4, SOX2 and CDX2 illustrated that both the porcine PA and IVF blastocysts were of high quality for further experiments (Fig 1D–1F and S1 Fig).

**Table 2:** The development of porcine embryos.

Group	No. of Embryos	Cleaved (%)	Blastocysts (%)
PA	1931	56.71±3.41	29.6±2.88
IVF	1290	57.75±4.03	30.63±2.88

**Table 3:** The quality of in vitro-fertilized porcine blastocysts and parthenogenetic porcine blastocysts.

Group	No. of Blastocysts	Mean (±SEM) no. of nuclei			ICM/Total (%)	TE/Total (%)
		ICM	TE	Total		
PA	9	10.89±2.29	42.67±4.91	53.30±5.85^a^	20.38±3.04	79.62±3.04
IVF	17	18.00±3.03	53.24±4.47	71.24±5.48^b^	24.78±2.98	75.22±2.98

In any particular row, values with different superscripts (a, b) indicate that the numbers are significantly different (P<0.05).

### The morphology, immunocytochemistry analysis and other characteristics of pTR cells

**Fig 2:**
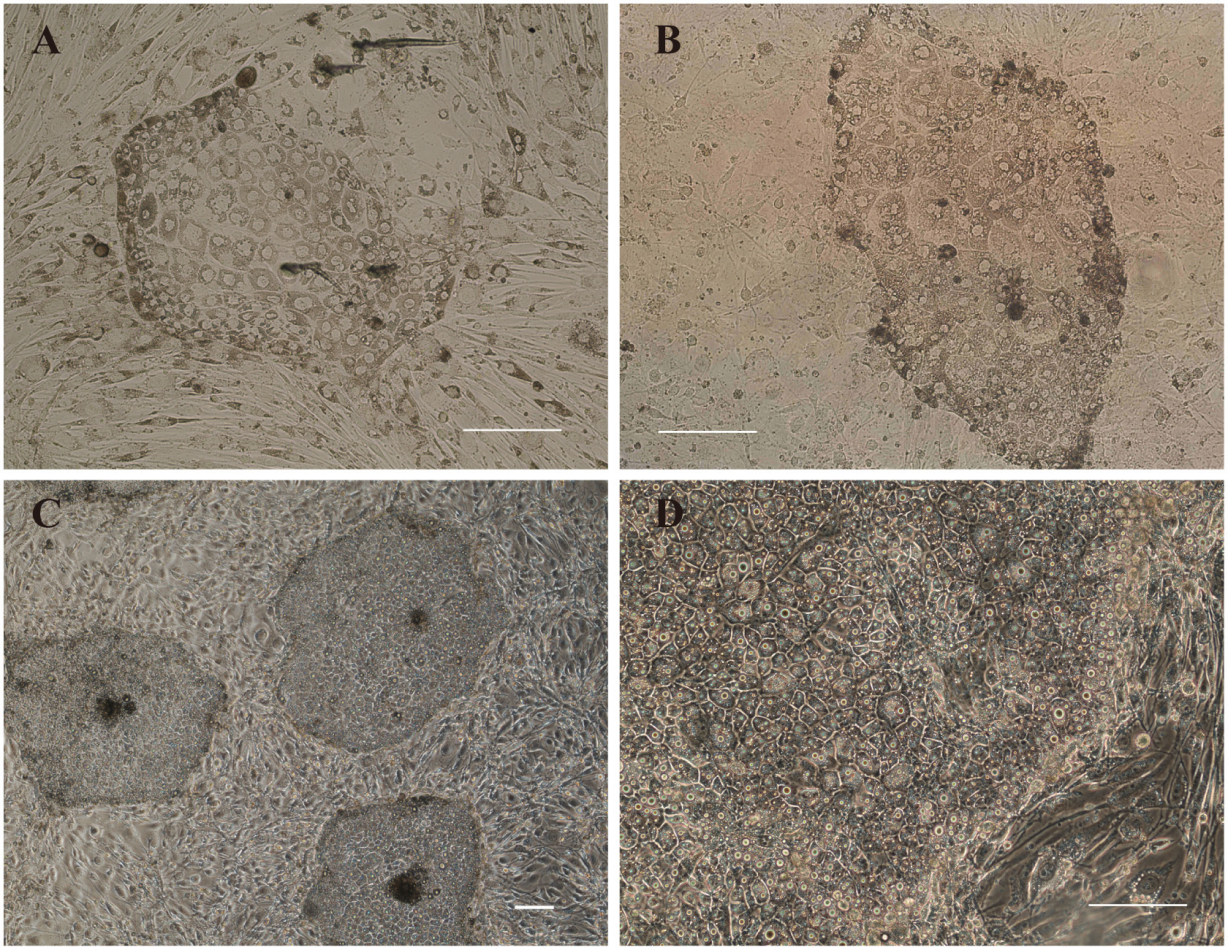
Derivation and morphology of porcine trophoblast cell. (A) Outgrowth of IVF blastocyst after 7 days of culture. (B) Outgrowth of PA blastocyst after 7 days of culture. (C-D) Morphology of pTR cell colonies at different magnification. All scale bars represent 200μm.

To derive trophoblast cells, both day 6–7 IVF and PA blastocysts were transferred to the KOSR/bFGF and STO feeder cells culture plates. The blastocysts floated in the culture medium at the beginning, a few blastocysts contracted and attached to the feeder cells approximately 3 days later, while the others remained in suspension and died. The primary outgrowths appeared within 2–5 days after the embryos attached and expanded gradually (Fig 2A and 2B). In the process of deriving TR cells, the IVF blastocysts showed higher adherence ratio than PA blastocysts (53.02±3.36 vs. 43.35±7.03, P>0.05), but the outgrowth ratio was similar between adherent IVF and PA blastocysts (46.97±9.60 vs. 44.57±6.05). The percentage of cell line derivation for IVF embryos is also higher than that of PA embryos (82.14±8.99 vs. 58.09±14.74, P>0.05) (Table 4). 11 cell lines from 14 IVF outgrowths and 11 cell lines from 27 PA outgrowths passaged over 2 times. The 11 cell lines grown from IVF embryos were named pIVFTR 1–11, and the 11 cell lines grown from PA embryos were named pPATR 1–11. Out of those, 2 pIVFTR cell lines and 5 pPATR cell lines were passaged more than 20 passages. One of the cell lines (pPATR-5) survived with routine passaging every 7 days at a dilution of approximately 1:6 for 110 passages (i.e., >700 days). In general, IVF outgrowths grew and survived better than PA outgrowths, although their morphology had no apparent differences. Both IVF and PA derived cells exhibited epithelial-like morphology, dark cytoplasmic and clear boundaries between cells (Fig 2A and 2B), and contained a significant amount of lipid droplets (Fig 2C and 2D), these characteristics were different from flat human embryonic stem cells and the cells growth from ICM. Additionally, some cells presented with multiple nuclei, and developed clear boundaries with the feeder cells. The cell line pPATR-5 became more uniform in appearance with each passage.

**Table 4:** The derivation of porcine trophoblast cells.

Group	No. of Blastocysts	Adhered (%) (no.)	Outgrowths (%) (no.)	TR cell lines * (%) (no.)
PA	145	43.35±7.02 (59)	44.57±6.05 (28)	58.09±14.74 (11)
IVF	54	53.02±3.36 (32)	46.97±9.60 (14)	82.14±8.99 (11)

* TR cell lines represent the cells passaged over 2 generations.

**Fig 3:**
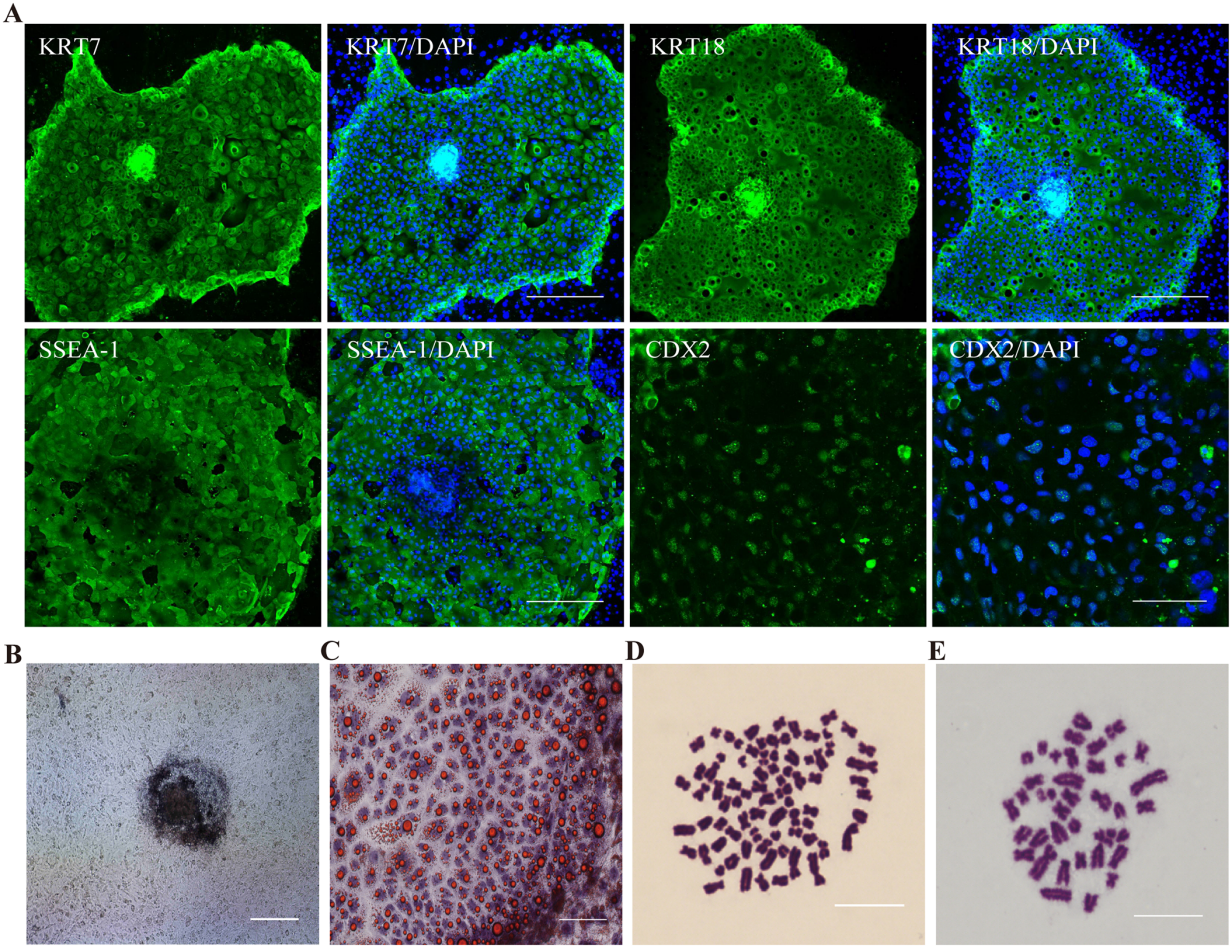
Characteristic of the pTR cells. (A) Immunofluorescence staining exhibited pTR cells (pPATR-5) is SSEA1, CDX2, KRT18 and KRT7 positive. There is no difference between IVF and PA pTR cells (S2 Fig). The scale bar represents 100μm (B) Alkaline phosphatase staining of pTR cells is positive. The scale bar represents 200μm. (C) Oil red staining of pTR cells (pPATR-5) showed the abundant lipid droplets. The scale bar represents 200μm. (D-E) Chromosome analyses of pTR cells. pPATR-5 is tetraploid (chromosome number is 76) showed in D, pIVFTR-7 is diploid (chromosome number is 38) shown in E. The scale bar represents 5μm.

The pTR cells derived from porcine PA and IVF blastocysts were not only similar in morphology, but also in immunocytochemistry analysis. The cells stained positively for KRT7, KRT18, SSEA1 and CDX2 (Fig 3A, S2 and S3 Figs). The TR marker KRT7 and KRT18 were detected in the cytoplasm of all cells (Fig 3A, S2 and S3 Figs). KRT7, which is generally regarded as a diagnostic TR marker, expressed with particularly high levels in the cells near the edges of the pTR cell colonies. Staining for trophectoderm marker CDX2, which has been detected in blastocysts, was positive in all cells and co-localized with nuclei (Fig 3A, S2 and S3 Figs). Positive staining of these markers confirmed the pTR cells had the characteristics of trophoblast cells. However, the pluripotency markers OCT4 and SOX2 which were positively stained in blastocysts (Fig 1D–1F and S1 Fig) could not be detected in pTR cells. Feeder layer STO cells were negative for CDX2, KRT7 and KRT18. Another similar feature of pPATR cells and pIVFTR cells was that they both exhibited positive staining of pluripotency marker SSEA1 (Fig 3A, S2 and S3 Figs) and AP (Fig 3B), even the AP staining was weak. Other pluripotency markers such as NANOG, SSEA4, TRA-1-60 and TRA-1-81 were also stained negatively (S4 Fig). The accumulation of the lipid droplets in the cytoplasm was obvious and can be dyed by Oil Red O (Fig 2C).

### Chromosome number analysis of pTR cells

Chromosomal analysis was performed on 4 pPATR cell lines and 3 pIVFTR cell lines. Of the 4 pPATR cell lines, pPATR-5 and pPATR-7 appeared to have a tetraploid karyotype with 76 chromosomes (Fig 3D); pPATR-2 and pPATR-6 were abnormal with the chromosome numbers varying from 38 to 76; moreover, euploid cells were rarely observed. Interestingly, the pPATR-2 and pPATR-6 cells were diploid at early stages of culture and doubled to a tetraploid or became abnormal at later stages. Of the 3 pIVFTR cell lines, chromosome number of pIVFTR-6 and pIVFTR-7 were 38 (Fig 3E), which is the normal number of chromosomes for pig cells; however, the chromosome number of pIVFTR-4 was abnormal.

### Gene expression analysis of pTR cells

**Fig 4:**
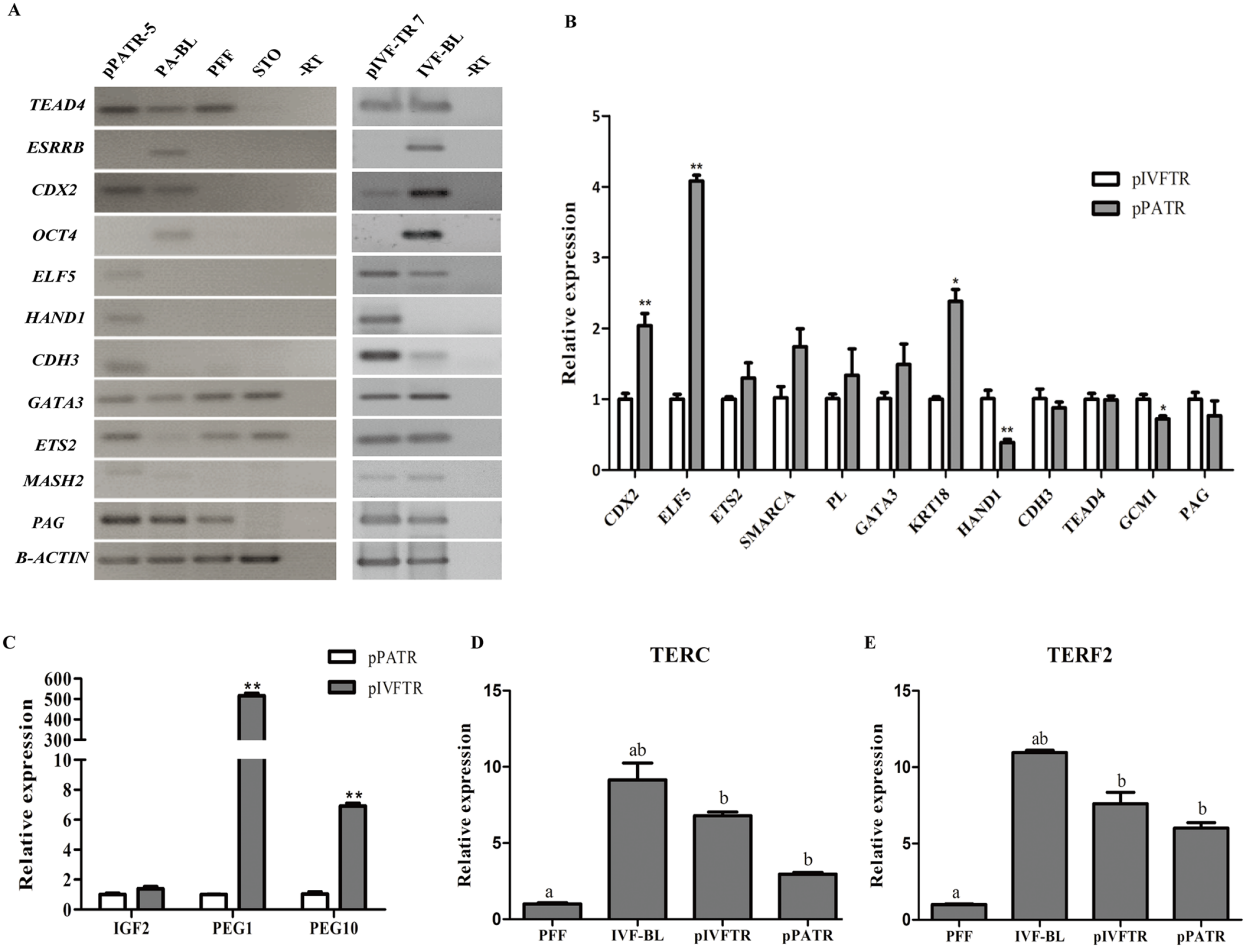
Genes expression analysis of pTR cells. (A) RT-PCR analysis of trophoblast and pluripotency genes in pTR cells. The expression of TSC and TR markers such as *CDX2*, *TEAD4*, *ELF5*, *HAND1*, *CDH3*, *GATA3*, *EST2*, *MASH2* and *PAG* was detected in pPATR-5 and pIVFTR-7, and *ESRRB* was not detected in both pTR cells but in blastocysts. The pluripotentcy marker *OCT4* is only expressed in blastocysts. *β-ACTIN* gene is used as control. (B) Real-time PCR analysis of trophoblast genes in pTR cells. The genes including *CDX2*, *ELF5* and *KRT18* are expressed significantly higher in pPATR-5 than in pIVFTR-7. The gene, *HAND1* and *GCM1* are expressed significantly higher in pIVFTR-7 than in pPATR-5. (C) Real-time PCR results of imprinted genes in pTR cells. Three paternally imprinted genes, *IGF2*, *PEG1* and *PEG10*, were expressed lower in pPATR-5 than in pIVFTR-7. (D-E) Real-time PCR results of telomerase activity-related genes in pTR cells. *TERC* and *TERF2* had significantly higher expression in pPATR-5 and pIVFTR-7 than in PFF. *β-ACTIN* is used as an endogenous control for trophoblasts genes in B) and imprinted genes in C), and *GAPDH* is for genes, *TERC* and *TERF2*. PFF: porcine fetal fibroblast; STO: feeder cells; BL, blastocysts. Error bars indicate ± SEM. The * indicates P < 0.05 and the ** indicates P < 0.001. ^a,b^ indicates a statistically significant difference from any another group (P<0.05).

The results of reverse transcription-PCR showed that the trophoblast genes *TEAD4*, *CDX2*, *GATA3*, *ETS2*, *MASH2* and *PAG* were expressed in pIVFTR and pPATR cells and blastocysts; whereas *ELF5* and *CDH3* were detected in pPATR cells, pIVFTR cells and IVF blastocysts, but not in PA blastocysts. *HAND1* and *CDH3* were only expressed in pTR cells; moreover, *ESRRB* and *OCT4* were only detected in blastocysts (Fig 4A). In order to find the differences of gene expression between pPATR and pIVFTR cells, we quantified the relative mRNA abundance of several trophoblast and pluripotency-related genes in pPATR-5 and pIVFTR-7. Expression of genes for transcription factors *CDX2* [33–35], *ELF5* [36, 37], *ETS2* [37–40], *GATA3* [41–43] and *SMARCA4* [44–46], which are associated with trophoblast lineage emergence, differentiation and implantation, unique TR marker *KRT18* and placental lactogen (*PL)* were higher in pPATR-5 than in pIVFTR-7 (Fig 4B). The expression level of trophectoderm-special marker *CDX2* was highly expressed in pPATR-5 (>2-fold, P<0.01). Trophoblast-enriched *ELF5* and *KRT18* transcripts were also significantly expressed at a higher level in pPATR-5 (>4-fold, P<0.01 and >2-fold, P<0.05, respectively). *ETS2*, *GATA3*, *SMARCA4* and *PL* expressed about 2-fold higher in pPATR-5 and showed no significant differences. By contrast, other trophoblast genes, e.g. *HAND1*, *CDH3*, *TEAD4*, *GCM1* and *PAG* gene, expressed higher in pIVFTR-7 than in pPATR-5 (Fig 4B). Out of those, the expression of *HAND1* and *GCM1* showed significant differences (>2-fold, P<0.01 and >1-fold, P<0.05, respectively). However, the pluripotent gene *NANOG* and *SOX2* showed extreme low expression levels in both pPATR-5 and pIVFTR-7. To investigate the expression of imprinted genes in pPATR and pIVFTR cells, three paternally imprinted genes (*IGF2*, *PEG1* and *PEG10*) were investigated by real-time PCR. The expression levels of *PEG1* and *PEG10* were significantly lower in pPATR cells than in pIVFTR cells, but the expression level of *IGF2* was no significantly difference in the two types of cell lines (Fig 4C). To investigate the telomerase activity of derived TR cells, we examined the expression levels of telomerase-related transcripts (*TERC* and *TERF2*) in pPATR and pIVFTR cells by real-time PCR. IVF blastocysts and PFF were used as positive and negative control, respectively. The telomerase RNA component *TERC* and the transcript of primary shelterin protein *TERF2* had significantly higher expression in pTR cells compared to PFF (Fig 4D and 4E).

### The ROCK inhibitor Y-27632 promotes the growth of pTR cells

**Fig 5:**
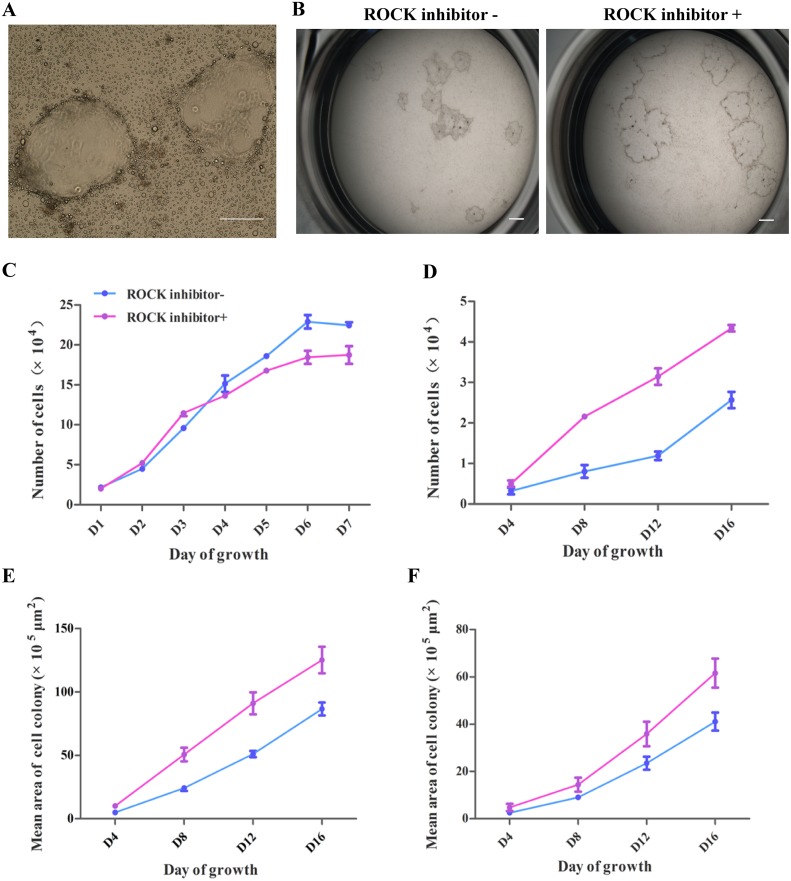
ROCK inhibitor Y-27632 on the morphology and growth of porcine trophoblast cells. (A) Dome structure formed after cultured with Y-27632. The scale bar represents 200μm. (B) The difference of pTR cell (pPATR-5) colony area with or without Y27832 for 8 days. (C-F) The growth curve of pTR cells cultured with or without Y-27632. C) The growth of PFF is suppressed by Y-27632, cells are decreased in numbers after culture 4 days. D) pPATR-5 cells that cultured in the present of Y-27632 are significantly improved. E) The mean colony area curve of pPATR-5 shows similar trend with the cell number curve. F) The mean colony area curve of pIVFTR-7. Y-27632 promoted the growth of both pIVFTR cells and pPATR cells.

Two best grown cell lines pIVFTR-7 and pPATR-5 were used for testing the effect of ROCK inhibitor. To make the passaging process more efficient and labor-saving, we tempted to digest the pTR with trypsin to replace the mechanical method. Unexpectedly, the proliferation of pTR cells decreased significantly after trypsinized into single cell. The digested pTR cultures could not form large enough colonies for mechanical dissection. However, this situation was improved by the addition of the ROCK inhibitor Y-27632 for 48 hrs. Moreover, we found that the cell growth rate was significantly accelerated when Y-27632 was present during the entire culturing process, and cells could be continuously passaged at a dilution of 1:3. A similar growth boost phenomenon was observed for the undigested cells. The ROCK inhibitor Y-27632 promoted cell colonies growth. If the colonies were not passaged, they could reach several millimeters in diameter, and began to form dome-like structures (Fig 5A), which was not observed when colonies were cultured without Y-27632. The growth rate of pPATR-5 in the presence of Y-27632 was significantly increased compared with the control group (P<0.05). The doubling time of pPATR-5 in the first 4 days decreased from approximately 38 hrs to 22.5 hrs after Y-27632 added (Fig 5D). We also measured the colony area (Fig 5B) every 4 days using NIS-ELEMENT 3.2 software. The average area of pPATR-5 cell colonies also significantly increased in the group treated with Y-27632 (Fig 5E). As the duration of culture increased, the increased cell colony area and the increased cell population followed the same trend (Fig 5D and 5E). Therefore, we evaluated the pIVFTR cells growth rate only by measuring the colony area. As with pPATR-5, the average area of Y-27632 treated pIVFTR-7 cells significantly increased than the control group (Fig 5F). The effect of Y-27632 on other cells was also investigated. The proliferation rate of PFF cells, which was evaluated by counting the number of cells every day for 7 days, was decreased in the presence of Y-27632 (Fig 5C).

### The effect of ROCK inhibitor Y-27632 on genes expression of pTR cells

**Fig 6:**
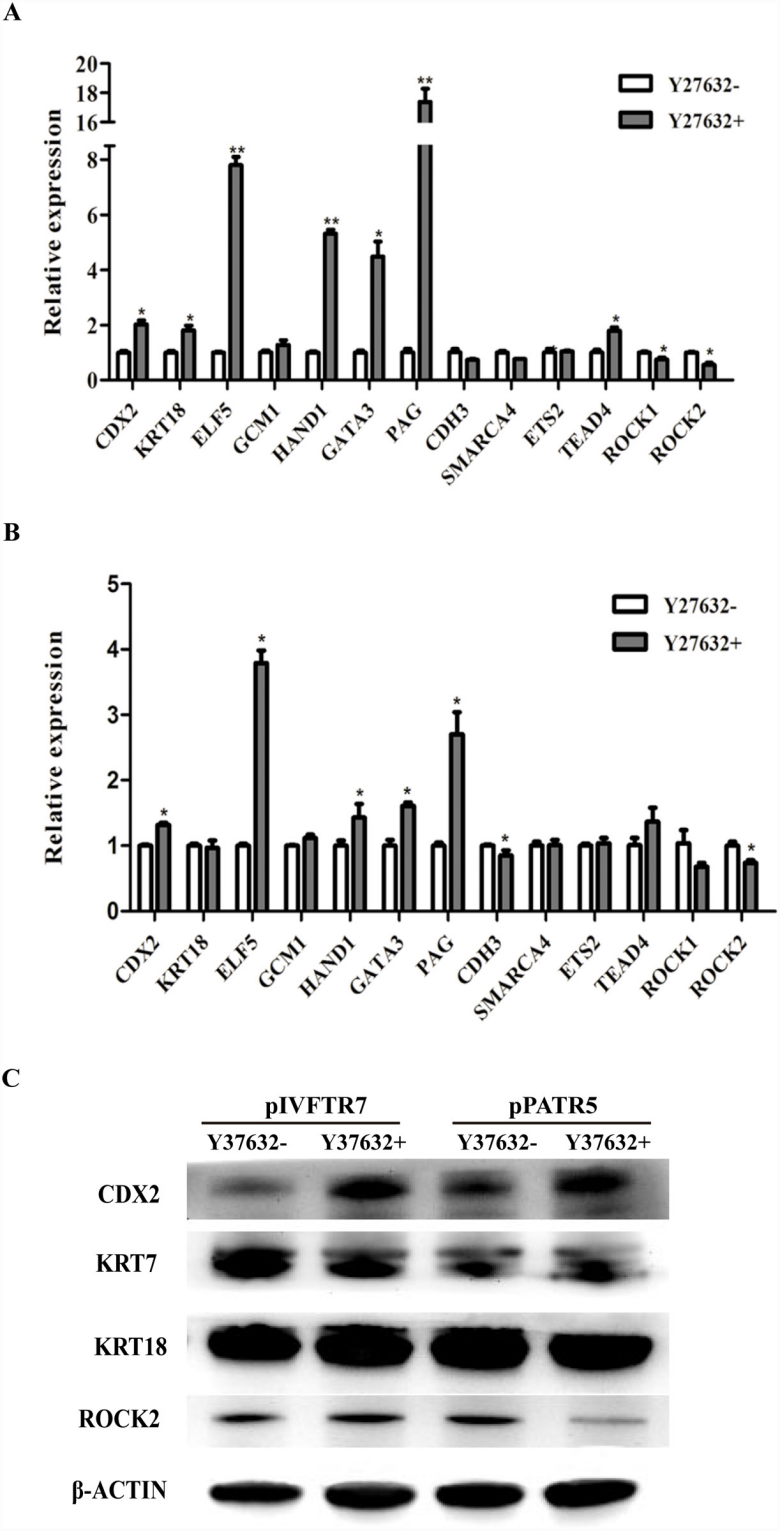
ROCK inhibitor Y-27632 on the gene expression of porcine trophoblast cells. (A-B) The real-time PCR analyses of gene expression changes of pTR cells cultured with Y-27632. Most trophoblast genes analyzed are up-regulated in Y-27632 treated cells (pIVFTR-7 A) and pPATR-5 B)). ROCK1 and ROCK2 are down-regulated. (C) Western blot detected proteins changes pTR cells cultured with Y-27632. CDX2 was remarkably increased in Y-27632 treated pIVFTR-7 and pPATR-5 cells. pTR cells cultured without Y-27632 in the same condition are used as control. β-ACTIN serves as an endogenous control. The * indicates P < 0.05 and the **indicates P < 0.001.

To further investigate the effect of Y-27632 on pTR cells, we first analyzed the trophoblast genes expression in pPATR-5 and pIVFTR-7 via quantitative real-time PCR. The expression of genes, such as *CDX2*, *ELF5*, *HAND1*, *GATA3* and *PAG* were significantly increased in pPATR and pIVFTR cells after Y-27632 supplement (Fig 6A and 6B). *TEAD4* was significantly increased in pIVFTR cells (Fig 6A), and *CDH3* was significantly decreased in Y-27632 cultured pPATR cells (Fig 6B). *ROCK1* and *ROCK2* were down-regulated by Y-27632 (Fig 6A and 6B). Western blot analysis of CDX2, KRT7, KRT18 and ROCK2 were performed on both Y-27632 treated and untreated pTR cells. CDX2 remarkably increased after treated by Y-27632, and ROCK2 levels decreased (Fig 6C). These results confirmed the gene expression changes indicated by quantitative real-time PCR. However, Y-27632 had little effect on KRT18 protein expression (Fig 6C), and KRT7 was decreased in Y-27632 treated pIVFTR cells but the change in pPATR cells was not obvious (Fig 6C).

### The ability of TR cells to differentiate into mature TR cells

**Fig 7:**
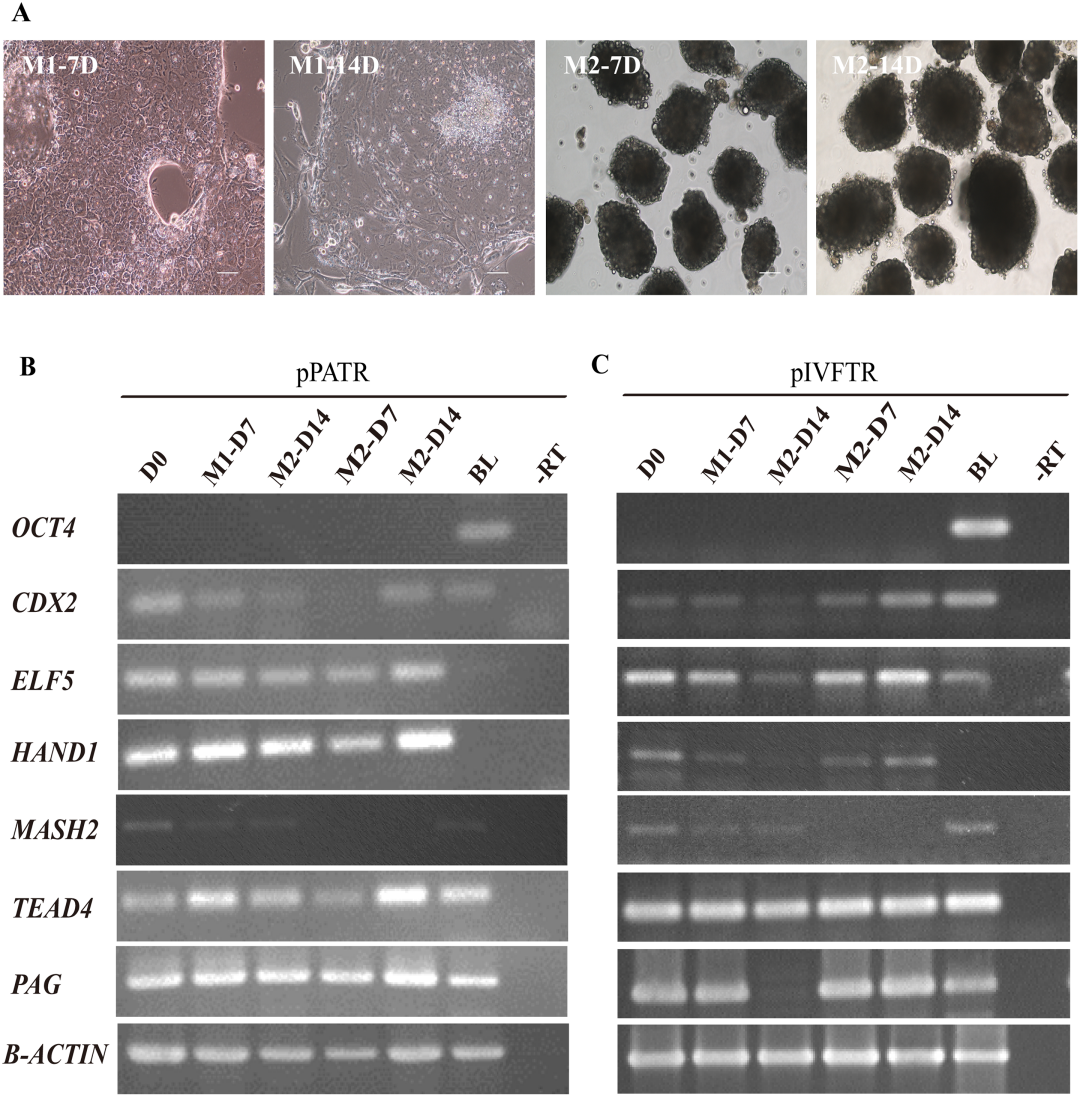
Culture pTR-E Cells in Different Medium. (A) Adherent cells grown in DMEM supplemented with 10% FBS (M1) at day 7 and day 14. Embryonic bodies like structures formed when bFGF removed from KF medium (M2) at 7 days and 14 days. The scale bar represents 100μm. (B-C) RT-PCR analyses of pTR cells cultured with different medium. *CDX2*, *ELF5*, *HAND1* and *TEAD4* were still expressed after differentiation. *MASH2* was not expressed in the matured cells cultured in M2. *PAG* in pIVFTR cells was absent after differentiation in the DMEM/F12/FBS medium for 14 days.

To investigate the differences in maturation potential of these blastocysts derived cells, pTR cells were cultured in two kinds of differentiation medium for 14 days after mechanically passaging. In the FBS containing DMEM medium, the majority of cells adherent to culture plate, and the oil droplets reduced, some cells formed cystic embryonic bodies like balls (Fig 7A). In the DMEM/F12 medium supplement with KOSR but without bFGF, the cells formed cystic embryonic body-like structures (Fig 7A). The genes, such as *CDX2*, *ELF5*, *HAND1* and *TEAD4*, were still expressed in two kinds of differentiated cells after differentiation culture (Fig 7B and 7C). However, the gene *MASH2*, which was expressed in the pTR cell, was not expressed in the matured cells cultured in the DMEM/F12/KOSR medium (Fig 7B and 7C). Similarly, the expression of *PAG* in pIVFTR cells was null after differentiation in the DMEM/F12/FBS medium for 14 days (Fig 7C).

## Discussion

In this study, the significant development difference between PA embryos derived by electrical activation and IVF embryos during *in vitro* culture was not observed. The PA embryos did not develop as well as IVF embryos, and the expression of OCT4, SOX2, and CDX2 in PA embryos was no obvious difference from IVF embryos. Porcine oocytes activated by electrical stimulation tended to generate high proportion haploid and diploid embryos [15]. However, only a limited number of authentic haploid cells could be obtained from porcine cleavage-stage PA embryos. These haploid cells had unstable karyotype, long-term culture of parthenogenetic embryos, *in vivo* or *in vitro*, resulting in abnormal chromosome numbers [15]. The diploid embryos derived TR cells also had unstable karyotype and resulted in abnormal chromosome numbers. Chromosome instability in PA embryos occurred at the cleavage and embryogenesis stages [47], and this instability might be due to the lack of a centriole which is inherited through the sperm in mammalian species, with the exception of rodents [48].

Chromosomal abnormalities are prevalent in aborted human [49], mouse [50], and pig conceptuses [51], moreover abnormal karyotypes are detected in nearly 30% of spontaneously aborted embryos [49]. In this study, one out of three IVF trophoblast cells exhibited abnormal chromosome numbers. A few reports addressed the chromosome abnormalities of porcine PA embryos [8]. In our previous report, the results showed the PA fetuses had smaller body sizes compared with IVF fetuses, and the chromosomal numbers of the PA fetus derived from fetal fibroblasts varied from 11 to 76 [15]. Although the same phenomena were observed from the PA derived trophoblast cell lines in this study, some of the pPATR cell lines had a stable tetraploid karyotype with over 60% cells containing 76 chromosomes. These tretraploid pPATR might be a useful tool to investigate and improve porcine somatic nuclear transfer efficiency through tetraploid compensation technology [52].

The trophoblast derived from pig *in vivo* embryos possess the capacity to spontaneously grow in culture in the absence of any immortalization procedure, and reach high passage numbers while retaining its major potentials [21], however, detailed characterization of PA embryos derived trophoblast cells, and comparison with IVF derived trophoblast cells has not been reported. Although the PA blastocyst had smaller size (data not shown), however, its TE/Total ratio was higher than IVF blastocysts. The derivation and characterization of trophoblast will provide the alternative material to study placental development and chromosome instability of PA embryos. In the present study, both IVF and PA blastocyst were used to derive trophoblasts, and total 22 outgrowths were passaged over twice, but only 4 PA and 3 IVF trophoblast cell lines were performed for chromosomal number analysis. The IVF blastocysts tended to attach and grow more easily, but during the routine passaging process the cell appearance and the growth ability of PA trophoblast cells were much better than IVF counterpart.

All pTR cell lines derived in this study exhibited trophoblast characteristics. Intermediate filament cytokeratin-7 (KRT7) is highly expressed throughout the trophoblast lineage [53, 54]. It has been used to assess the purity of human placental villous trophoblast cells by flow cytometry [55]. KRT7 has been used as a marker to characterize the by-product trophoblastic cells during the standard reprogramming of porcine fibroblasts to pluripotent stem cells [22]. KRT18 is also solely expressed in the trophectoderm, and can therefore be used as a marker for trophectodermal differentiation [56–58]. Our results showed the trophoblast cells derived from porcine PA and IVF blastocysts were KRT7, KRT18 positive with no obvious differences between them. Additionally, these cells were rich in lipid droplets, which indicated that these cells had the typical characteristics of a trophoblast. At the same time, the pluripotency marker such as OCT4, NANOG, SSEA4, TRA-1-60 and TRA-1-81 were negative as assayed by immunofluorescent analysis, which implied these cells were different from embryonic stem cells. SSEA1 was stained positively in pTR cells, which has historically been used as a marker of pluripotent stem cells, including embryonic stem cells, and was first described on blastomeres of the 8 cell stage mice embryo and transient expressed in trophectoderm [59]. Populations of SSEA1+ cells have recently been identified in the adult, including in the bone marrow, spleen, heart, and brain [60]. The expression of SSEA1 in porcine trophoblast needs the further experiment to confirm.

CDX2 and OCT4 plays pivotal roles in the segregation of the ICM and TE during the development of mouse embryos [33, 34, 61], which has been used as a specific marker that is capable of distinguishing between TE and ICM cells in mice and in other animals [34, 35]. However, *CDX2* and *OCT4* transcripts were detected in both the ICM and TE in porcine and bovine blastocyst embryos [62–64]. The *CDX2* level in porcine ovoid embryos was significantly higher in the TE than in the ED (embryonic disc, ED). Thus, the differences in the CDX2/OCT4 ratio between ED and TE cells became clearer during development from the blastocyst to elongated stage. This pattern of *OCT4* and *CDX2* expressions observed in porcine embryos is similar to that of bovine embryos [63, 64]. CDX2 was detected in bovine trophoblast stem cells at both the transcription and protein levels [65], which resembles its expression in mouse as undifferentiated trophoblast stem cells [66], but coexpressed with *OCT4* [65]. In this study, the expression of CDX2 in PA and IVF pTR cells was confirmed by RT-PCR, immunofluorescence and Western blot analysis, but the expression of pluripotency marker OCT4 was negative. The expression of *OCT4* was detected in early passage cells but its expression was very low, and diminished after 2–3 passages (data not shown), which indicated that our culture system cannot sustain the OCT4 expression and the derived cells has the typical characteristics of trophoblast. The transcription factor *ELF5* was an Ets-superfamily member characterized by a pointed and winged helix DNA binding domain, essential to extraembryonic ectoderm maintenance as shown by loss of function mutants in which the embryonic ectoderm directly adjoins an ectoplacental cone (EPC)-like layer of trophoblast [67] and necessary for correct trophoblast development [68]. *ELF5* and *CDX2* along with *EOMES*, *TAFP2*, *ETS2*, *ESRRB*, *SOX2* and *FOXD3* involved the gene regulatory network involving mouse trophoblast stem cell maintenance [37]. *ELF5* is expressed later than *CDX2*, that is, from expanded blastocyst stages [36], a role of *ELF5* in preventing precocious differentiation of trophoblast stem cells by enhancing stem-like genes such as *SOX2*, and inhibiting expression of trophoblast differentiation markers. The expression of *ELF5*, *CDX2* and other trophoblast stem cell genes such as *TEAD4*, *ETS2*, *GATA3* and *SMARCA4* in pPATR and pIVFTR cells indicated the cells derived in this study were at the early stage of development. At the same time, the different expression of trophoblast genes, imprinted genes *PEG1* and *PEG10*, and telomerase activity related genes *TERC* and *TERF2* indicated the TR cells derived from IVF and PA blastocysts were not exactly identical, the further study need to be conducted to illustrate the reasons of these differences.

Y-27632 is known as a highly selective ROCK inhibitor [27, 28], plays a role in trophoblast cell migration, cell polarity determining and epithelial mesenchymal transitions [69]. The presence of 20 μM Y-27632 increased the rate of attachment and trophoblast differentiation from hESCs as evidenced by the anti-apoptotic, anti-detachment and pro-survival activity [31]. In this study, the addition of the ROCK inhibitor Y-27632 improved the pTR cell growth rate both in dissociated cells and in undisturbed colonies and increased the expression of trophoblast marker gene such as *CDX2*, *ELF5*, *HAND1*, *GATA3*, *GCM1*, *TEAD4* and *PAG*. This suggests that Y-27632 exactly enhanced the pTR cells vitality and trophoblast characteristics. Therefore, Y-27632 could be used to improve the efficiency of pTR derivation from blastocysts. The differential expression patterns of trophoblast genes suggest ROCK inhibitor Y-27632 may have a positive effect on pTR cells and the effect on pIVFTR and pPATR cells was different. In addition, pTR cells survival time was prolonged with the present of Y-27632, i.e. pPATR-7 was passaged over 40 generations in the present of Y-27632, but only reached 25 generations in the KF medium (data not shown). The growth of all cell lines in Y-27632 cultivation was superior to KF. However, the mechanism of the ROCK inhibitor Y-27632 effect on pig TR cells and the functions of ROCK1 and ROCK2 in pig TR cells require further study.

In summary, our data demonstrated that the porcine trophoblast cells can be obtained from both parthenogenetic and IVF embryos. Our results suggested that there are intrinsic differences between the two types of TR cells. The gene expression pattern and the ability to develop into mature trophoblast cells indicated that the trophoblast cells established in this study are not only at the early stage of development, but also have the characteristics of trophoblast stem cells. These trophoblast cells may serve as a useful model for studying porcine trophoblast development *in vitro*.

## Supporting Information

S1 Fig Immunofluorescence staining of pluripotency markers in porcine PA blastocysts. (A) OCT4. (B) CDX2. (C) SOX2. The scale bar represents 50μm.
(TIF)

S2 Fig Immunofluorescence staining of SSEA1, CDX2, KRT18 and KRT7 in pIVFTR cells (pIVFTR-7). The scale bar represents 100μm.
(TIF)

S3 Fig Immunofluorescence staining of KRT7, KRT18, SSEA1 and CDX2 in other pPATR or pIVFTR cell lines. (A) pPATR-7 cell line. (B) pIVFTR-6 cell line. The scale bar represents 100μm.
(TIF)

S4 Fig Immunofluorescence staining of pluripotency markers in porcine TR cells. (A-C) The staining results of OCT4 on both TR cells and feeder layers. The same antibody was used to stain porcine blastocysts, which indicated the pTR cells are OCT4 negative. (D-F) TRA-1-81 and (G-I) TRA-1-60 immunofluorescence staining in pTR cells. DAPI is used to label the nuclei, bright field is used to identify cell colony. The scale bar represents 200μm. (J-K) SOX2 and (L-M) NANOG staining were negative. The scale bar represents 50μm.
(TIF)
