# Supplementary figures and images for: The Efficient Derivation of Trophoblast Cells from Porcine In Vitro Fertilized and Parthenogenetic Blastocysts and Culture with ROCK Inhibitor Y-27632
Source: PLoS One. 2015 Nov 10;10(11):e0142442. doi: 10.1371/journal.pone.0142442 (PMC4640852; doi:10.1371/journal.pone.0142442)

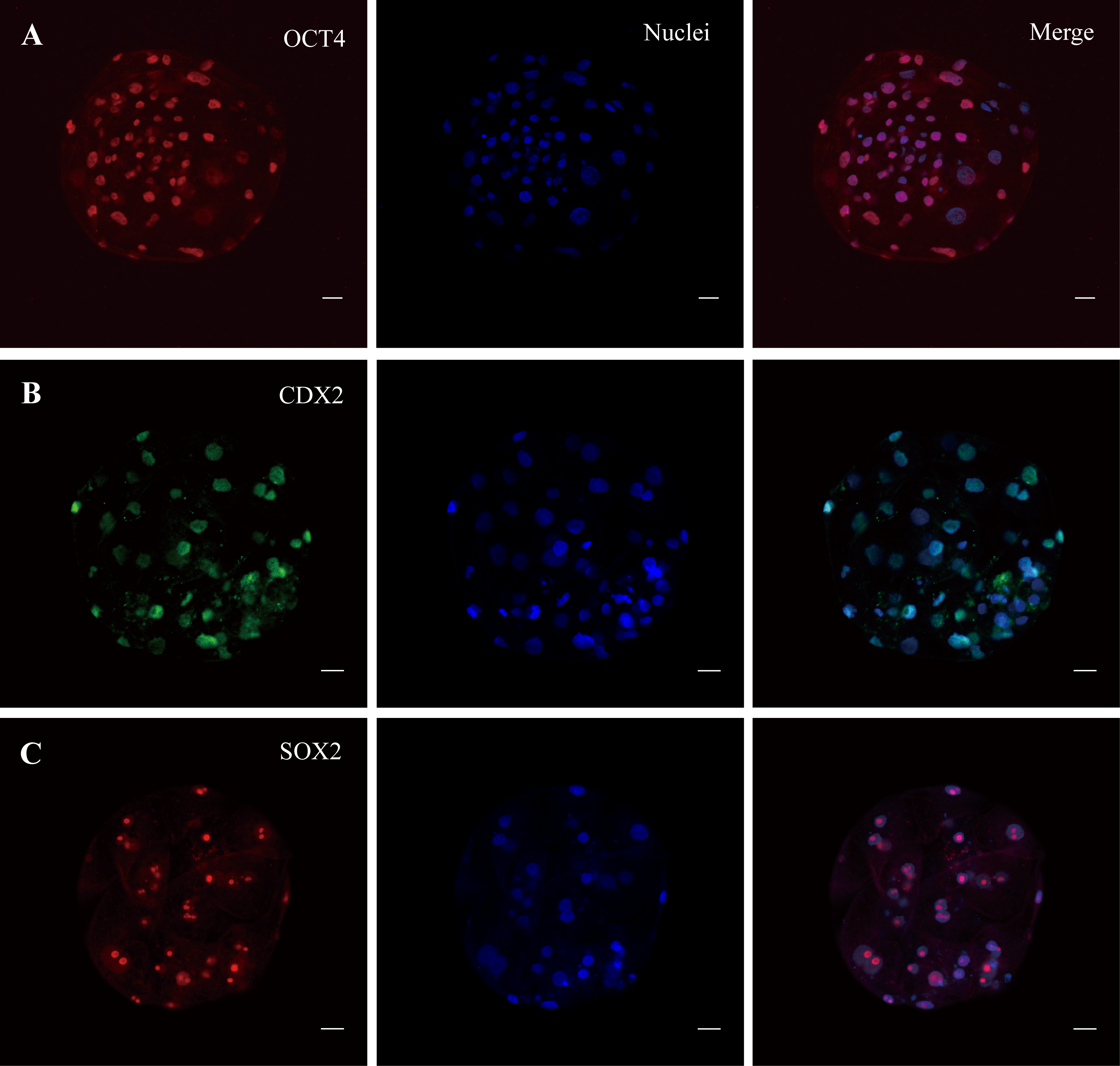

Supplement: S1 Fig — (A) OCT4. (B) CDX2. (C) SOX2. The scale bar represents 50μm. (TIF) [file pone.0142442.s001.tif]

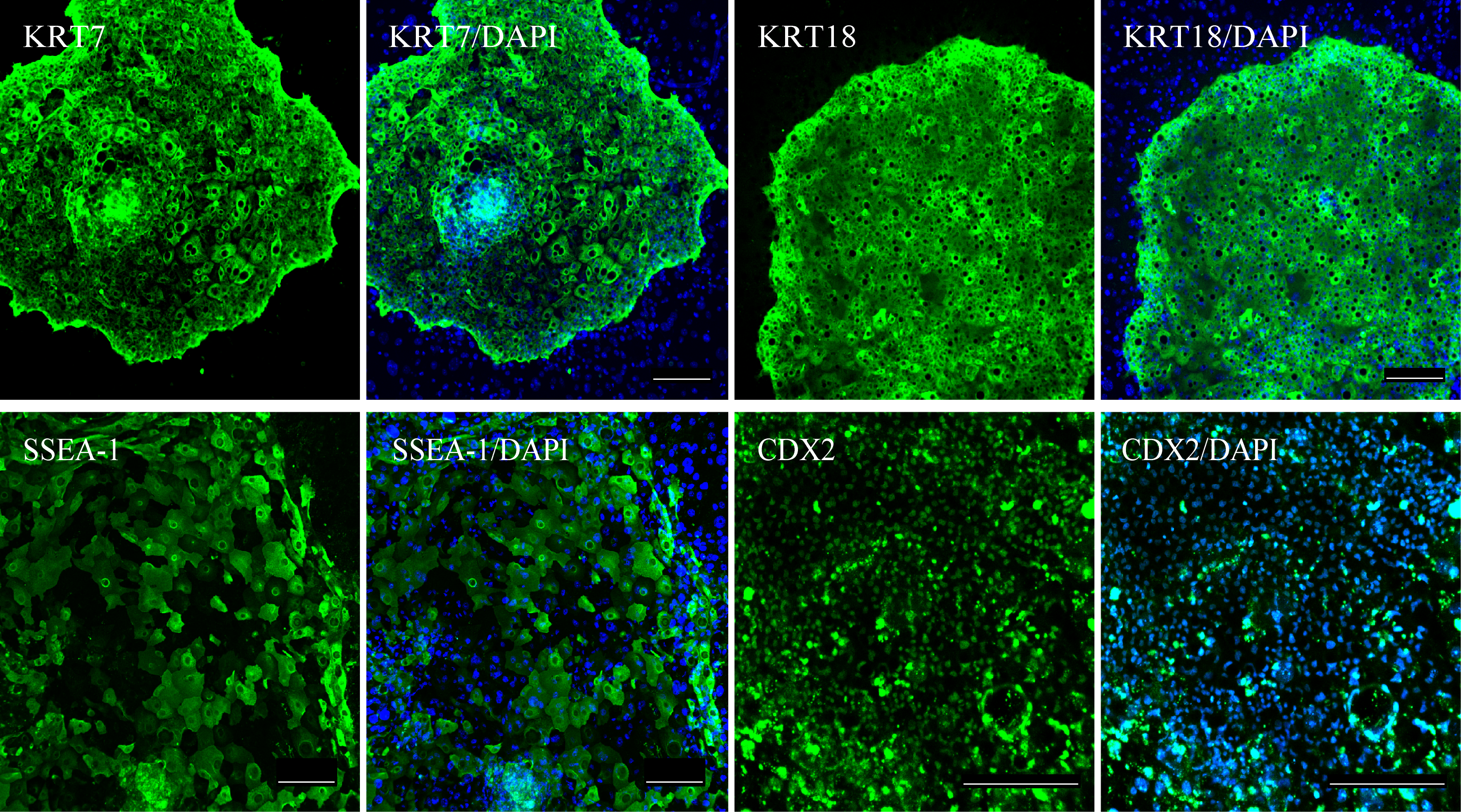

Supplement: S2 Fig — The scale bar represents 100μm. (TIF) [file pone.0142442.s002.tif]

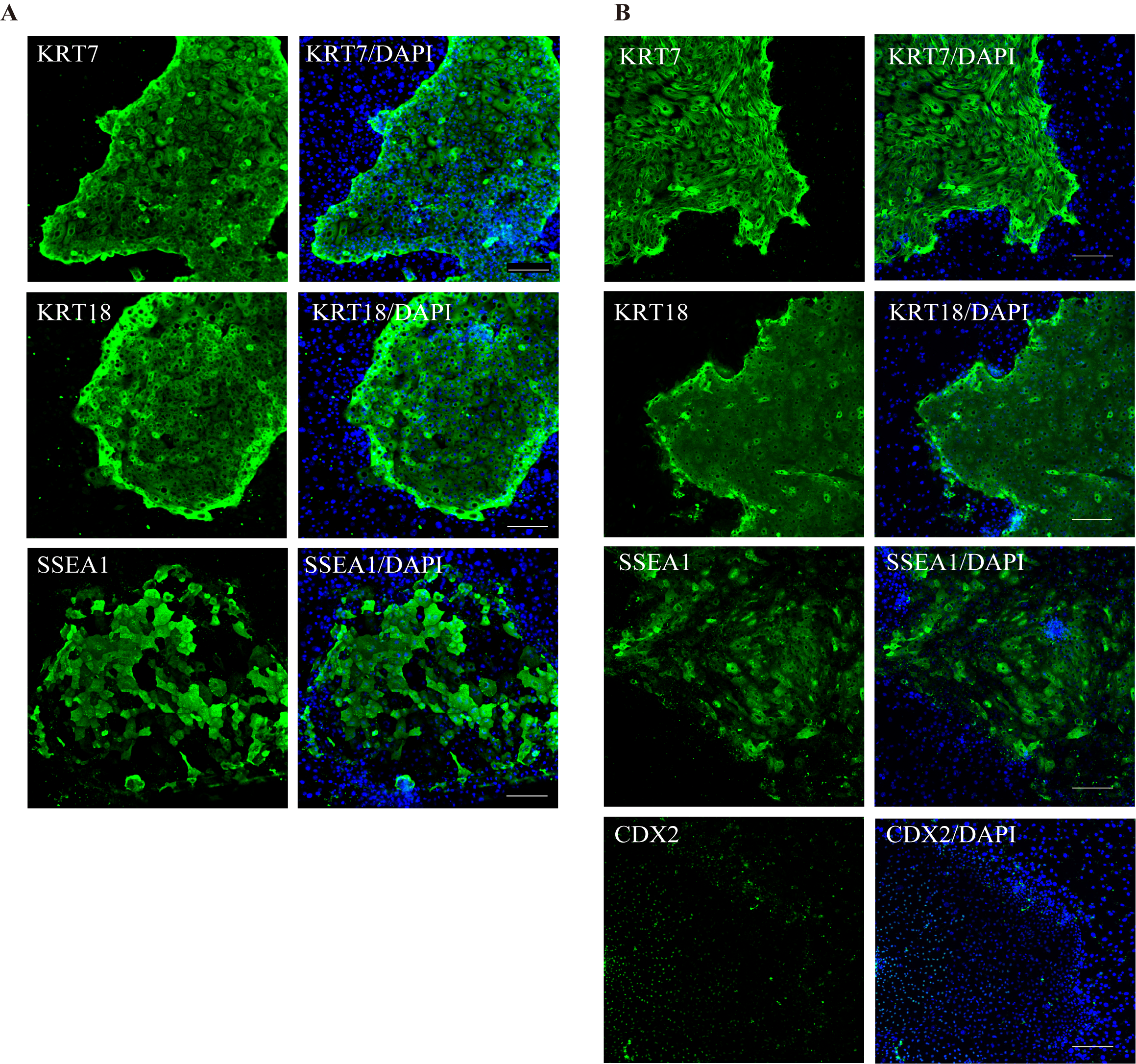

Supplement: S3 Fig — (A) pPATR-7 cell line. (B) pIVFTR-6 cell line. The scale bar represents 100μm. (TIF) [file pone.0142442.s003.tif]

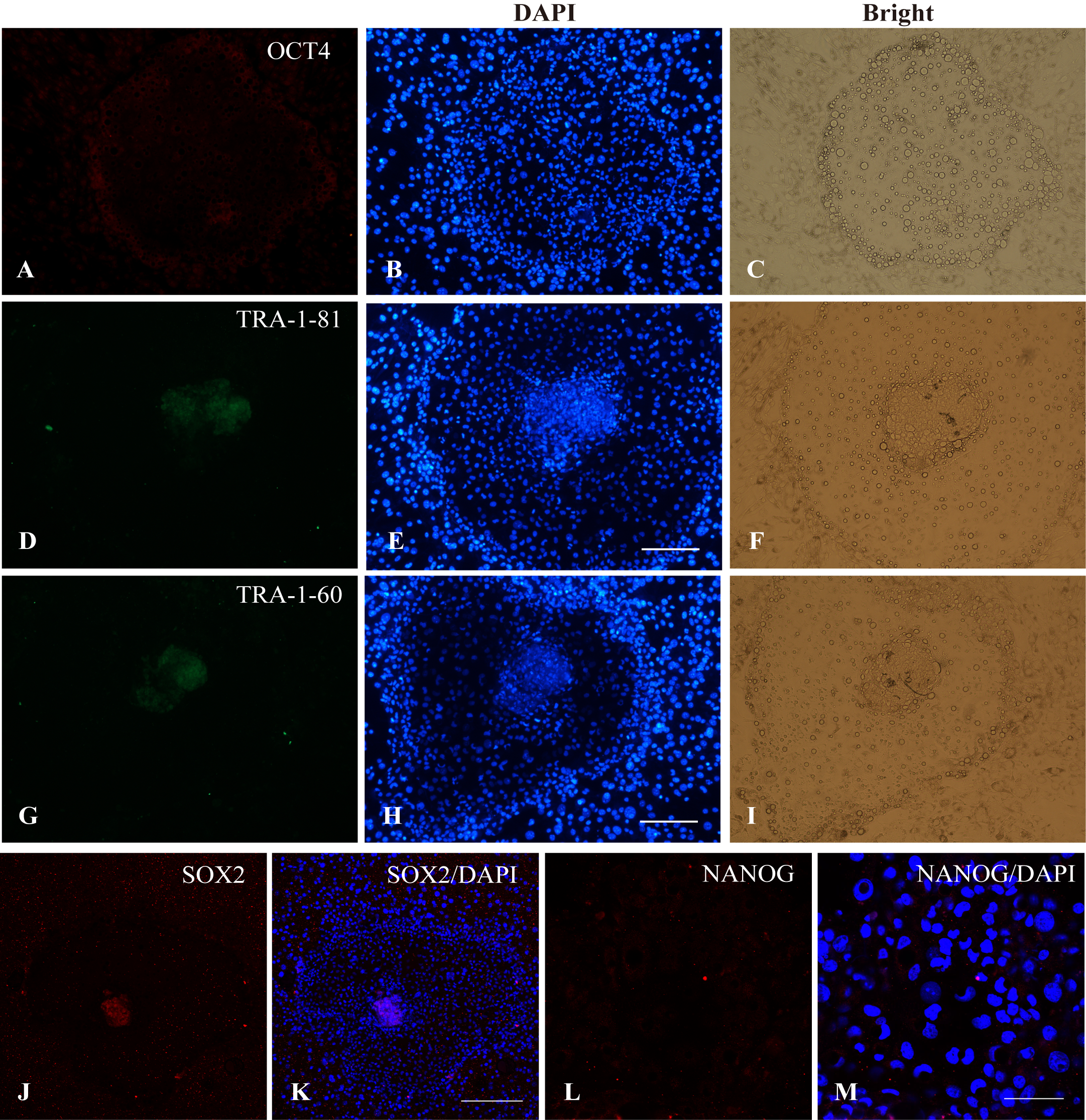

Supplement: S4 Fig — (A-C) The staining results of OCT4 on both TR cells and feeder layers. The same antibody was used to stain porcine blastocysts, which indicated the pTR cells are OCT4 negative. (D-F) TRA-1-81 and (G-I) TRA-1-60 immunofluorescence staining in pTR cells. DAPI is used to label the nuclei, bright field is used to identify cell colony. The scale bar represents 200μm. (J-K) SOX2 and (L-M) NANOG staining were negative. The scale bar represents 50μm. (TIF) [file pone.0142442.s004.tif]
